# CEUS with VEGFR2-targeted microbubbles for monitoring of early immunotherapy effects in a colorectal cancer model

**DOI:** 10.1186/s40644-026-01101-0

**Published:** 2026-07-31

**Authors:** Felix L. Herr, Jonathan K. Stock, Verena Gräfin zu Solms-Baruth, Larissa V. Blume, Sandra Kloiber-Langhorst, Heidrun Hirner-Eppeneder, Jennifer Stueckl, Barbara Akla, Jens Ricke, Thomas Geyer, Wolfgang Kunz, Maurice M. Heimer, Dirk-Andre Clevert, Melissa J. Antons, Clemens C. Cyran

**Affiliations:** 1 Department of Radiology, LMU University Hospital Munich, Marchioninistr. 15, 81377 Munich, Germany; 2 Bracco Suisse SA, Plan-les-Ouates, Switzerland

**Keywords:** Contrast-enhanced ultrasound, VEGFR2-targeted microbubbles, Immunotherapy monitoring, Colorectal cancer, Tumor angiogenesis

## Abstract

**Background:**

To evaluate contrast-enhanced ultrasound (CEUS) with VEGFR2-targeted microbubbles for longitudinal monitoring of early treatment effects during combined anti-PD-L1/anti-CTLA-4 immunotherapy in a murine colorectal cancer model.

**Methods:**

Murine colorectal cancer allografts (CT26) were established subcutaneously in 29 female Balb/c mice (therapy *n* = 15; control *n* = 14). Baseline CEUS with VEGFR2-targeted microbubbles was performed on day 7. The therapy group received intraperitoneal anti-PD-L1 and anti-CTLA-4 antibodies between days 7–15; controls received sham treatment. Follow-up CEUS was performed on days 14 and 21. Tumor perfusion (WiAUC) and VEGFR2 binding (SI_8min_, SI_10min_) were quantified, and immunohistochemistry assessed CD8, Ki-67, TUNEL, CD31, and VEGFR2.

**Results:**

At FU1, WiAUC was significantly lower in the therapy group compared with controls (4,029 ± 61 vs. 6,391 ± 412; *p* < 0.001), and remained significantly lower at FU2 (1,028 ± 27 vs. 2,049 ± 39; *p* < 0.001). VEGFR2-targeted microbubble binding was consistently lower under therapy. At FU1, SI_8min_ was 407 ± 11 vs. 626 ± 13 and SI_10min_ was 409 ± 10 vs. 634 ± 28 (both *p* < 0.001). At FU2, SI_8min_ was 106 ± 6 vs. 207 ± 4 and SI_10min_ was 107 ± 5 vs. 207 ± 4 (both *p* < 0.001). Immunohistochemistry confirmed higher apoptosis and tumor-infiltrating lymphocytes, and lower proliferation, microvascular density, and VEGFR2 expression in treated tumors (all *p* < 0.05).

**Conclusions:**

VEGFR2-targeted CEUS enabled longitudinal monitoring of early response to dual anti-PD-L1/anti-CTLA-4 immunotherapy in colorectal cancer. Treated tumors showed lower perfusion and VEGFR2-targeted binding, paralleled by reduced vessel density and VEGFR2 expression as well as increased CD8 infiltration and apoptosis. These findings support VEGFR2-targeted CEUS as a promising noninvasive imaging biomarker for monitoring early immunotherapy-associated vascular changes.

**Supplementary Information:**

The online version contains supplementary material available at 10.1186/s40644-026-01101-0.

## Graphical Abstract

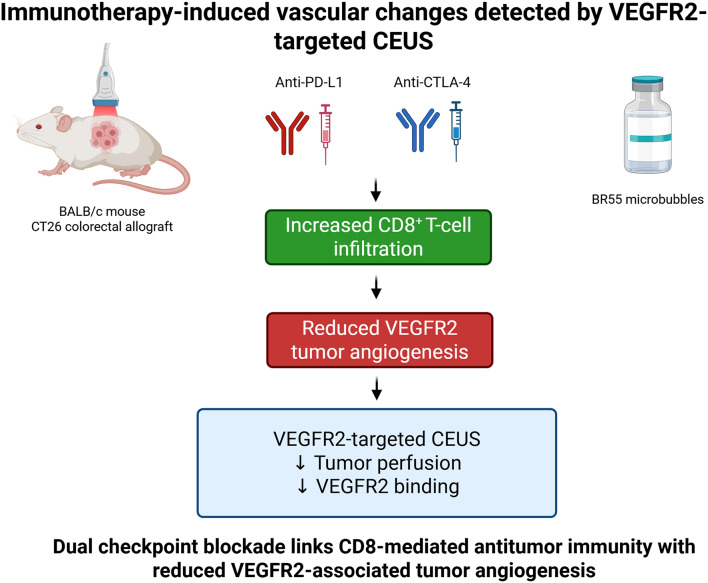

## Background

**Fig. 1:**
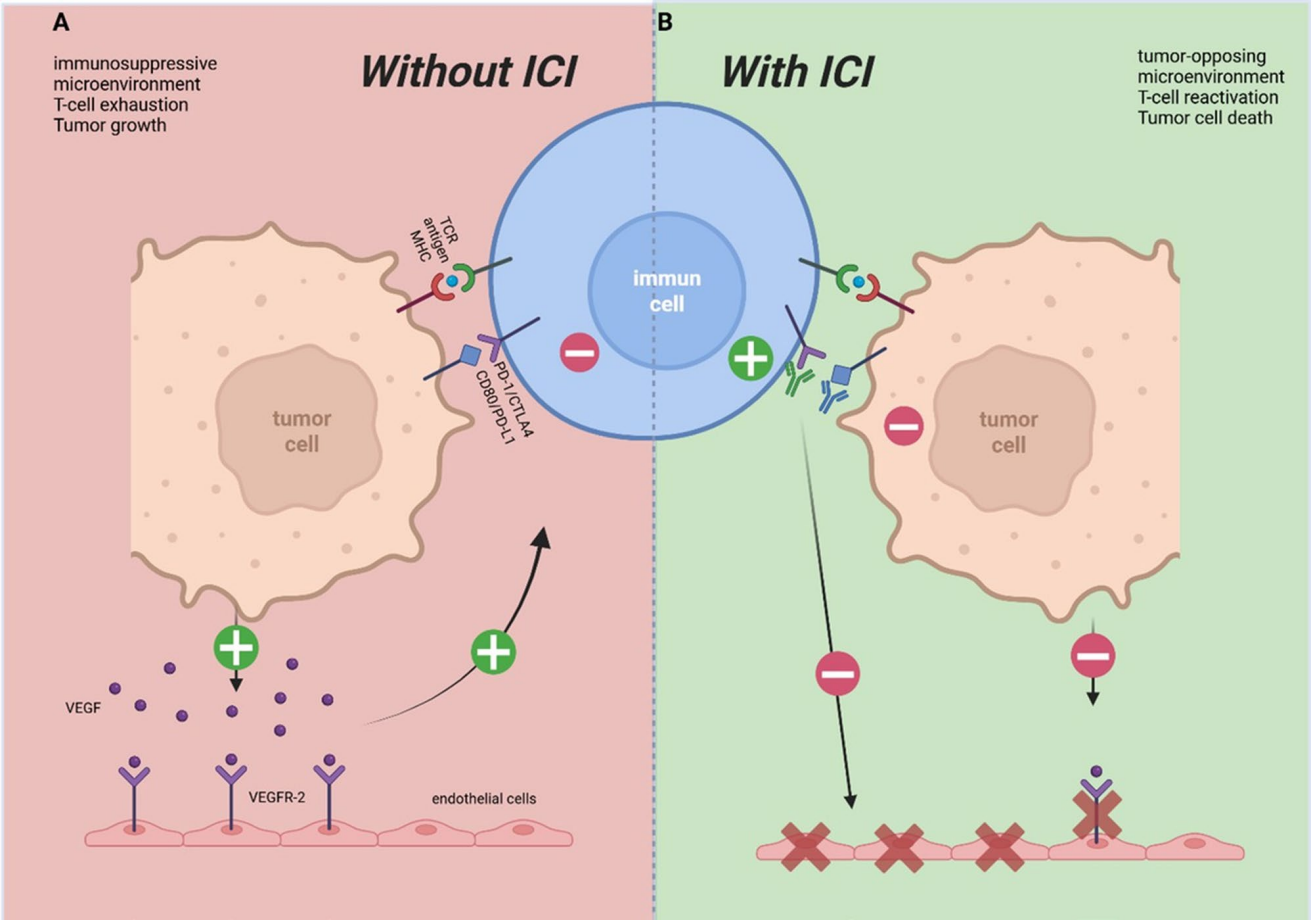
VEGF/VEGFR2 signaling and immune modulation in colorectal cancer without and with ICI. The left panel (Without ICI) depicts the immunosuppressive tumor microenvironment characterized by high VEGF/VEGFR2 signaling, aberrant angiogenesis, and impaired immune cell activity. VEGF released by tumor cells binds to VEGFR2 on endothelial cells, promoting vascular proliferation and contributing to T-cell exhaustion and tumor growth. The right panel (With ICI) shows the effects of dual checkpoint blockade (anti-PD-L1 and anti-CTLA-4), which inhibits immune-suppressive signaling, restores cytotoxic T-cell activity, and results in lower VEGFR2-mediated angiogenesis, resulting in vascular normalization, enhanced immune infiltration, and tumor cell death. Figure created with BioRender

Immune checkpoint inhibitors (ICI) have transformed cancer therapy but show limited efficacy in advanced colorectal cancer (CRC). Most CRCs are microsatellite stable (MSS) and exhibit a cold immune microenvironment characterized by poor T-cell infiltration [1, 2]. Only the small subset of mismatch repair-deficient, microsatellite unstable (MSI) CRCs, accounting for approximately 4–5% of cases, demonstrates responsiveness to ICI [3]. Although CT26 tumors are MSS, they remain immunologically responsive when sufficient T-cell priming is achieved, and robust CD8 T-cell activation can mediate tumor eradication in experimental settings [4]. This conditional immunogenicity makes CT26 a well-established MSS CRC model for immune profiling and preclinical evaluation of immunotherapeutic strategies in immunocompetent mice [5–7]. VEGF (vascular endothelial growth factor) and its receptor VEGFR2 drive tumor angiogenesis and contribute to immune suppression [8–10] (Fig. 1). Enhanced VEGF/VEGFR2 signaling promotes abnormal vasculature, hypoxia, and reduced immune cell infiltration, while impairing dendritic cell maturation and cytotoxic T-cell function and upregulating immune checkpoints such as PD-L1 [11–17] (Fig. 1A). This interplay between angiogenesis and immune evasion sustains tumor growth. ICI can partially disrupt this cycle by restoring CD8 T-cell activity and promoting vascular normalization [11, 18] (Fig. 1B).

In clinical immuno-oncology, objective tumor shrinkage often occurs late during immune checkpoint therapy [19]. However, therapeutic response remains heterogeneous, highlighting the need for early response biomarkers capable of capturing treatment-induced changes before volumetric alterations become apparent [20]. Functional imaging techniques that assess tumor perfusion and angiogenic remodeling may be particularly suited to detect early immunotherapy effects [21]. VEGFR2 represents a promising imaging target for monitoring tumor vasculature during ICI therapy, as it reflects active angiogenic signaling within the tumor endothelium. Contrast-enhanced ultrasound (CEUS) enables real-time, noninvasive assessment of perfusion and angiogenesis [22–25]. When combined with VEGFR2-targeted microbubbles, CEUS allows specific binding to intravascular endothelial VEGFR2 and visualization of angiogenic tumor vessels. Clinically, MSS colorectal cancers typically exhibit limited responsiveness to ICI monotherapy due to immune exclusion and low intrinsic immunogenicity. However, dual checkpoint blockade with anti-PD-L1 and anti-CTLA-4 can enhance T-cell priming and modulate the tumor microenvironment, thereby partially overcoming resistance mechanisms [26]. Although VEGFR2-targeted CEUS has previously been investigated for monitoring anti-angiogenic therapies, its utility for longitudinal assessment of therapy-associated vascular changes during immune checkpoint inhibition remains incompletely understood. It remains unclear whether VEGFR2-targeted CEUS can detect early vascular remodeling and angiogenic modulation induced by dual ICI before macroscopic tumor regression occurs. Therefore, the aim of this study was to evaluate whether longitudinal VEGFR2-targeted CEUS can noninvasively detect early immunotherapy-induced changes in tumor perfusion and angiogenic activity in MSS colorectal cancer.

## Methods

### Animal model and experimental protocol

All procedures complied with institutional and national regulations for laboratory animal care and were approved by the Committee for Animal Research of the Government of Upper Bavaria (ROB-55.2-2532.Vet_02-24-81). The study followed ARRIVE guidelines. Female Balb/c mice (10–12 weeks, ~ 20 g) were housed four per cage under controlled conditions (22 ± 2 °C, 55 ± 10% humidity, 12-h light/dark cycle) with ad libitum access to food and water, standard bedding, and environmental enrichment. Humane endpoints included body weight loss ≥ 19%, tumor size > 1.5 cm, ulceration, infection, hemorrhage, diarrhea, apathy, ascites, or dermatitis.

CT26 murine colorectal carcinoma cells were injected subcutaneously (3 × 10⁵ cells) into the left flank. When tumors reached approximately 0.5 cm (day 7), mice were randomized by lottery to therapy (*n* = 15) or control (*n* = 14). The therapy group received intraperitoneal anti-PD-L1 (BioXCell, #BP0101) and anti-CTLA-4 (BioXCell, #BP0131) antibodies (20 mg/kg) on days 7, 9, 11, 13, and 15; controls received phosphate-buffered saline. For imaging, anesthesia was maintained with 2.0 vol% isoflurane and 1.5 L/min oxygen on a heating pad. CEUS was performed on days 7 (baseline), 14, and 21. At endpoint, animals were euthanized and tumors from an independent non-imaged cohort were collected for histological validation. This cohort underwent identical treatment and assessment schedules and was included to avoid potential effects of repeated CEUS, microbubble administration, and anesthesia on tumor biology.

### Study design overview

**Fig. 2:**
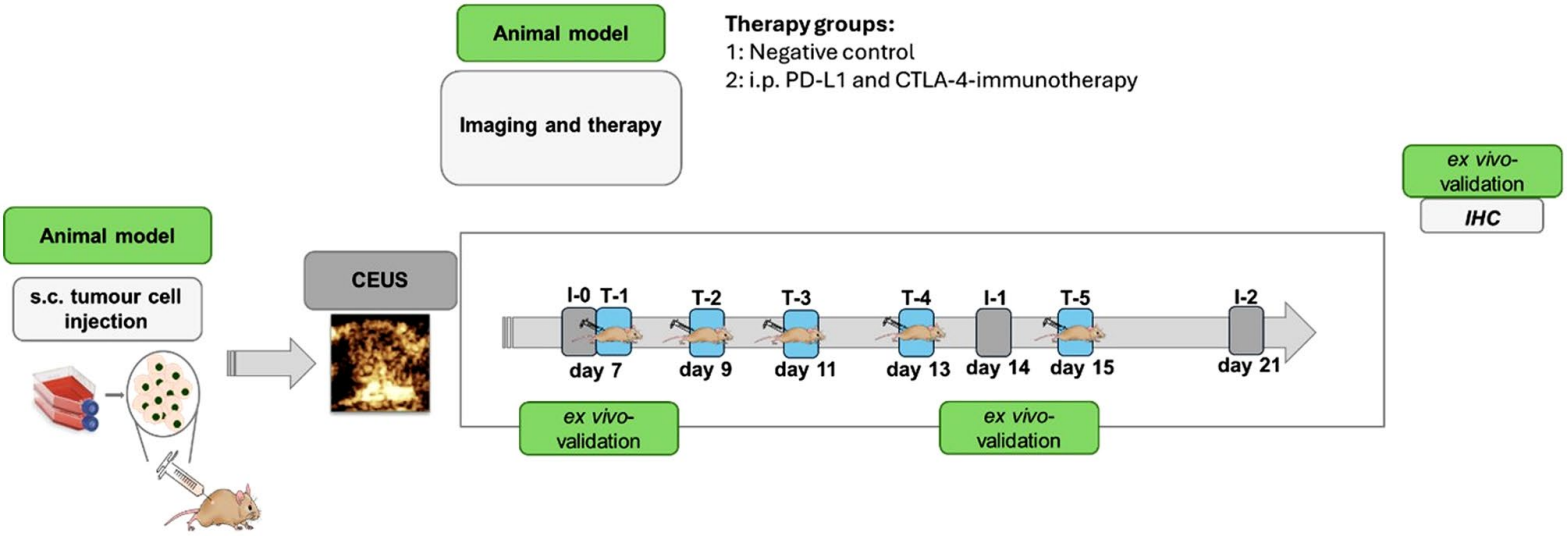
Experimental design. Following subcutaneous inoculation of CT26 MSS colorectal carcinoma cells into Balb/c mice, longitudinal CEUS imaging using VEGFR2-targeted microbubbles (BR55) was performed on days 7 (baseline), 14 (follow-up 1), and 21 (follow-up 2). In parallel, combined ICI with anti-PD-L1 and anti-CTLA-4 antibodies were administered intraperitoneally on days 7, 9, 11, 13, and 15 post-inoculation. At each imaging time point, ex vivo validation was performed through immunohistochemical analyses in an independent cohort of animals

In total, 50 female Balb/c mice were included in this study. 29 animals were allocated to the CEUS imaging cohort and randomly assigned to a therapy group (*n* = 15) or a control group (*n* = 14). Of these, 22 completed the full experimental protocol including longitudinal CEUS. Three animals from the therapy group and four animals from the control group were excluded due to anesthesia-related complications, or technical imaging failure. The therapy group received intraperitoneal anti-PD-L1 and anti-CTLA-4 antibodies on days 7, 9, 11, 13, and 15, while controls received vehicle. CEUS imaging was performed longitudinally at baseline (day 7) and follow-up on days 14 and 21. For histological validation, an independent cohort (*n* = 21; therapy *n* = 10, control *n* = 11) was analyzed under identical experimental conditions. Tumors were harvested at baseline, follow-up 1, and follow-up 2 for immunohistochemical and morphometric analyses. To minimize bias, investigators performing CEUS acquisition, CEUS image analysis, and histological quantification were blinded to treatment allocation. The study workflow is illustrated in Fig. 2.

### Microbubbles

BR55 (Bracco Suisse SA, Plan-les-Ouates, Switzerland) is a VEGFR2-targeted, lipid-shelled ultrasound contrast agent. A heterodimeric peptide ligand is conjugated to DSPE-PEG2000 and incorporated into the microbubble shell. The lyophilized agent contains a perfluorobutane–nitrogen gas mixture and is reconstituted with 2 mL of 5% glucose before use [24, 27, 28]. Microbubbles have a mean diameter of approximately 1.5 μm and a concentration of ~ 2 × 10⁹/mL [24, 29]. BR55 remains in circulation for approximately four minutes following intravenous administration [25]. For each imaging session, 200 µL of BR55 suspension was injected manually via a tail vein catheter under standardized conditions.

### CEUS imaging

CEUS imaging was performed using an EPIQ 7 ultrasound system (Philips Healthcare, Seattle, WA, USA) equipped with an 18L4 linear transducer (transmit frequency 9 MHz, dynamic range 55 dB, imaging depth 25 mm). Contrast-specific pulse inversion imaging was performed at a low mechanical index to detect microbubbles [24]. This diagnostic low-mechanical-index imaging protocol was used for microbubble detection and was not intended to induce vascular disruption or therapeutic bioeffects. Following BR55 administration, continuous image acquisition was performed for 1 min to assess wash-in kinetics, followed by intermittent acquisitions every 2 min up to 10 min post-injection (frame rate 16 Hz). Tumor perfusion was assessed from wash-in/wash-out kinetics, whereas VEGFR2-specific binding was evaluated at 8 and 10 min after injection, when circulating microbubbles had largely cleared from the bloodstream while retained targeted microbubbles remained bound to the tumor endothelium. No non-targeted control microbubble arm was included, as the approved animal protocol was designed for longitudinal treatment comparison and did not permit additional animal groups in accordance with the 3R principle.

### Data post-processing

All CEUS data were analyzed offline using dedicated quantification software (VueBox^®^, 7.5.0.7051; Bracco Suisse SA, Plan-les-Ouates, Switzerland). A region of interest (ROI) was manually placed within the viable, contrast-enhancing tumor rim. Necrotic regions, identified on B-mode and CEUS images as hypoechoic or heterogeneous intratumoral areas lacking contrast enhancement, were excluded from ROI placement. The software generated time-intensity curves (TICs) based on relative echo-power values reflecting microbubble concentration [24, 30, 31]. Wash-in area under the curve (WiAUC) was used as a surrogate marker of tumor perfusion and vascularization. For the late molecular phase, mean signal intensities at 8 (SI_8min_) and 10 min (SI_10min_) post-injection represented VEGFR2-specific binding. Tumor dimensions were measured on B-mode ultrasound images in two orthogonal planes. Tumor area (mm²) was calculated as the product of the maximum longitudinal and transverse diameters and used for longitudinal size assessment. These measurements were available only in animals undergoing longitudinal CEUS imaging. WiAUC was defined as the primary imaging endpoint, whereas SI_8min_, SI_10min_, and tumor area served as secondary endpoints.

### Histology and immunohistochemistry

Formalin-fixed, paraffin-embedded tumor sections were processed for immunohistochemistry. Serial 2-µm sections were deparaffinized and rehydrated. For CD8 and Ki67 staining, sections were permeabilized with 0.25% Triton X-100 in PBS. Antigen retrieval was performed using SignalStain^®^ EDTA Unmasking Solution (Cell Signaling Technology, Danvers, MA, USA) for CD8, CD31, and VEGFR2, or Universal HIER reagent (ab208572, Abcam, Cambridge, UK) for Ki67. Non-specific binding was blocked using Animal-Free Blocking Solution (Cell Signaling Technology) or bovine serum albumin in PBS. Sections were incubated overnight with anti-CD31 (1:100; ab28364, Abcam), anti-VEGFR2 (1:50; #2479, Cell Signaling Technology), anti-CD8 (1:100; ab217344, Abcam), and anti-Ki67 (1:50; SP6, MA5-14520, Thermo Fisher Scientific). Detection used goat anti-rabbit IgG (HRP) (1:400; ab150073, Abcam) and DAB+ chromogen (DAKO, Agilent Technologies). Slides were counterstained with hematoxylin and mounted. Microvascular density (CD31, VEGFR2) was quantified by counting positive microvessels in 10 randomly selected high-power fields (200× magnification). CD8 + and Ki67 + cells were quantified as the mean percentage of positive cells across 10 randomly selected fields. Histological quantification was performed by an investigator blinded to treatment allocation. Apoptosis was assessed by TUNEL staining (In Situ Cell Death Detection Kit, Fluorescein; Sigma-Aldrich) following heat-induced epitope retrieval. The apoptotic index was defined as the percentage of TUNEL-positive nuclei in 10 randomly selected high-power fields (200× magnification). For all quantitative analyses, fields were selected from viable tumor tissue. Areas of extensive necrosis, tissue-processing artifacts, and section borders were excluded from field selection. Necrotic area was quantified in H&E-stained sections using Fiji/ImageJ (NIH, USA). Ten randomly selected non-overlapping fields per tumor section were analyzed at 10× magnification. Necrotic regions were identified morphologically by loss of nuclear staining and eosinophilic cellular debris. The percentage of necrosis was calculated as necrotic area divided by total tissue area × 100. For each tumor, measurements from ten fields were averaged and used for statistical analysis.

### Statistical analysis

Data are expressed as mean ± standard deviation (SD). Between-group comparisons were performed using the Mann-Whitney U test. To control for multiple testing, p-values were adjusted using the Benjamini-Hochberg false discovery rate (FDR) procedure. A two-sided p value < 0.05 was considered statistically significant. No formal sample size calculation was performed. Group sizes were based on institutional experience with comparable experimental designs. All analyses were conducted using GraphPad Prism (version 10; GraphPad Software, Boston, MA) and Microsoft Excel (Microsoft Corporation, Redmond, WA).

## Results

### Tumor area

Tumor area was assessed longitudinally in the CEUS imaging cohort at baseline, follow-up 1, and follow-up 2 using two-dimensional B-mode ultrasound measurements. At baseline, there was no significant difference in mean tumor area between the therapy and control groups (14.1 ± 6.7 mm² vs. 15.3 ± 8.3 mm²; *p* = 0.889). At follow-up 1, tumor area increased in both groups (37.7 ± 25.4 mm² in the therapy group vs. 58.4 ± 34.3 mm² in controls), without reaching statistical significance (*p* = 0.068). At follow-up 2, tumor area further increased to 62.5 ± 36.5 mm² in the therapy group and 114.2 ± 50.9 mm² in controls, with a statistically significant difference (*p* = 0.015).

### CEUS

**Fig. 3:**
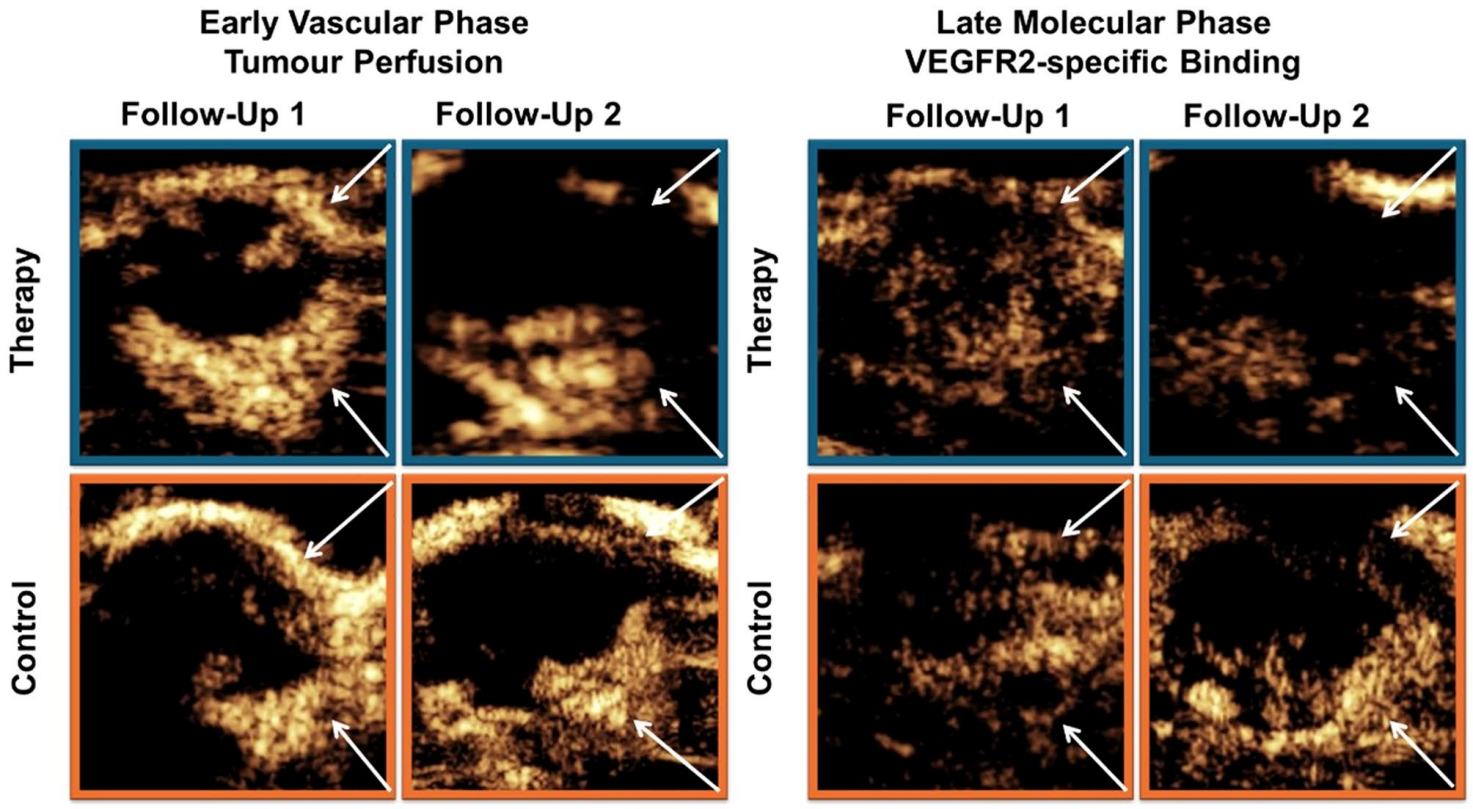
Representative BR55-targeted CEUS images of mice bearing subcutaneous tumor allografts under ICI. Therapy (blue) and control (orange) groups at follow-up 1, and follow-up 2. Arrows mark tumor allografts. The left panels show the early vascular phase reflecting functional tumor perfusion, whereas the right panels display the late molecular phase (10 min post-contrast injection) visualizing VEGFR2-specific microbubble binding. At follow-up 1 and 2, the therapy group demonstrated a significantly lower microbubble signal intensity during the early vascular phase compared with control, indicating lower tumor perfusion. Similarly, late-phase enhancement corresponding to VEGFR2-specific binding was significantly lower in the therapy group relative to control, consistent with therapy-induced vascular changes and lower VEGFR2 expression

**Fig. 4:**
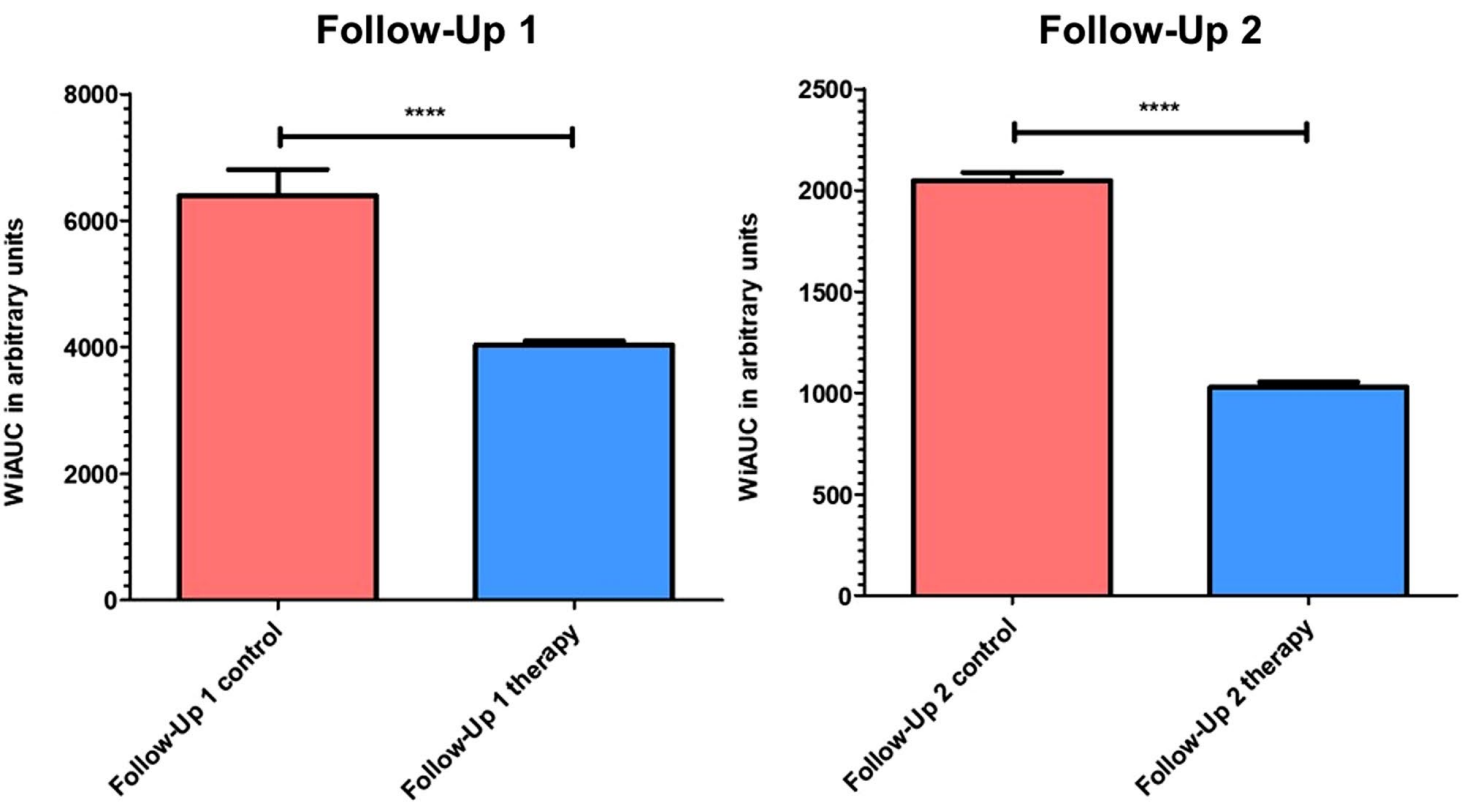
Column charts of WiAUC in therapy and control group at follow-up 1, and follow-up 2. Wash-in area under the curve (WiAUC) in arbitrary units for control (red, *n* = 10) and therapy (blue, *n* = 12) groups. WiAUC was significantly lower in the therapy group compared with controls at follow-up 1 (*p* < 0.001) and follow-up 2 (*p* < 0.001), indicating a significantly lower tumor perfusion under combined anti-PD-L1/anti-CTLA-4 treatment. Error bars represent mean ± standard deviation (SD)

**Fig. 5:**
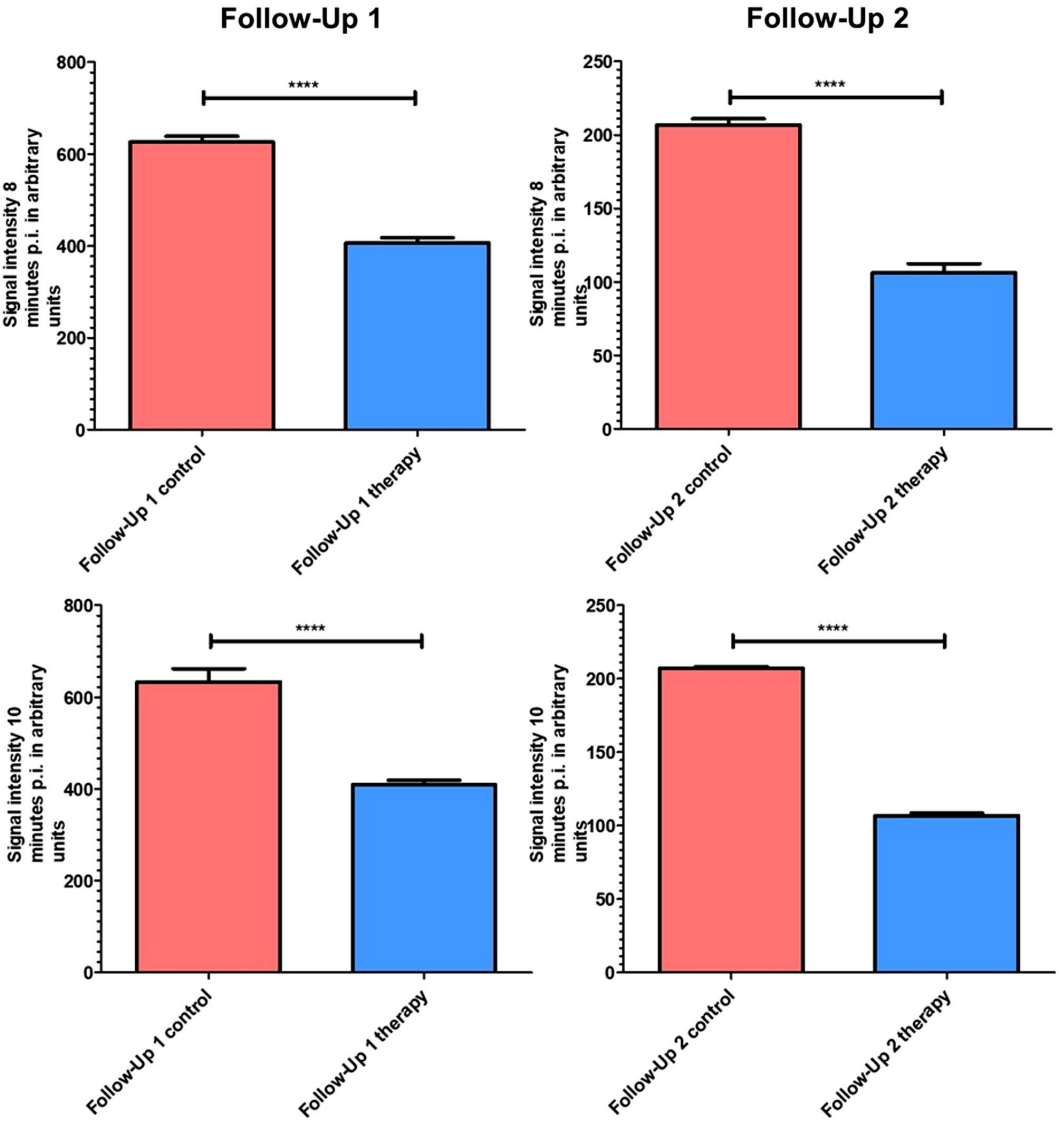
Column charts of SI_8min_ and SI_10min_ in therapy and control groups at follow-up 1, and follow-up 2. Signal intensities at 8 and 10 min post-contrast injection (SI_8min_ and SI_10min_, arbitrary units) are shown for the control (red, *n* = 10) and therapy (blue, *n* = 12) groups. At follow-up 1 and 2, both SI_8min_ and SI_10min_ were significantly lower in the therapy group compared with controls (all *p* < 0.001), indicating a significantly lower VEGFR2-specific microbubble binding and thus lower VEGFR2 expression following combined anti-PD-L1/anti-CTLA-4 therapy. Error bars represent mean ± standard deviation (SD)

Using BR55-targeted CEUS, tumor perfusion was quantified during the early vascular phase, and VEGFR2-specific microbubble binding was evaluated during the late molecular phase (Fig. 3). At baseline, no significant difference in perfusion was observed between therapy and control groups (WiAUC: 34,754 ± 4,543 vs. 33,570 ± 8,741; *p* = 0.710). At follow-up 1, WiAUC was significantly lower in the therapy group (4,029 ± 61) compared to controls (6,391 ± 412; *p* < 0.001). This persisted at follow-up 2 (1,028 ± 27 vs. 2,049 ± 39; *p* < 0.001; Fig. 4). At baseline, no significant difference in late-phase signal intensity was found between therapy and control groups for SI_8min_ (1,191 ± 205 vs. 1,174 ± 227 a.u.; *p* = 0.310) or SI_10min_ (1,107 ± 77 vs. 1,096 ± 75 a.u.; *p* = 0.740). At follow-up 1, both SI_8min_ and SI_10min_ were significantly lower in the therapy group compared with controls (SI_8min_ = 407 ± 11 vs. 626 ± 13 a.u.; *p* < 0.001; SI_10min_ = 409 ± 10 vs. 634 ± 28 a.u.; *p* < 0.001). Both parameters remained significantly lower in the therapy group at follow-up 2 (SI_8min_ = 106 ± 6 vs. 207 ± 4 a.u.; *p* < 0.001; SI_10min_ = 107 ± 5 vs. 207 ± 4 a.u.; *p* < 0.001; Fig. 5).

### Immunohistochemical analysis

**Fig. 6:**
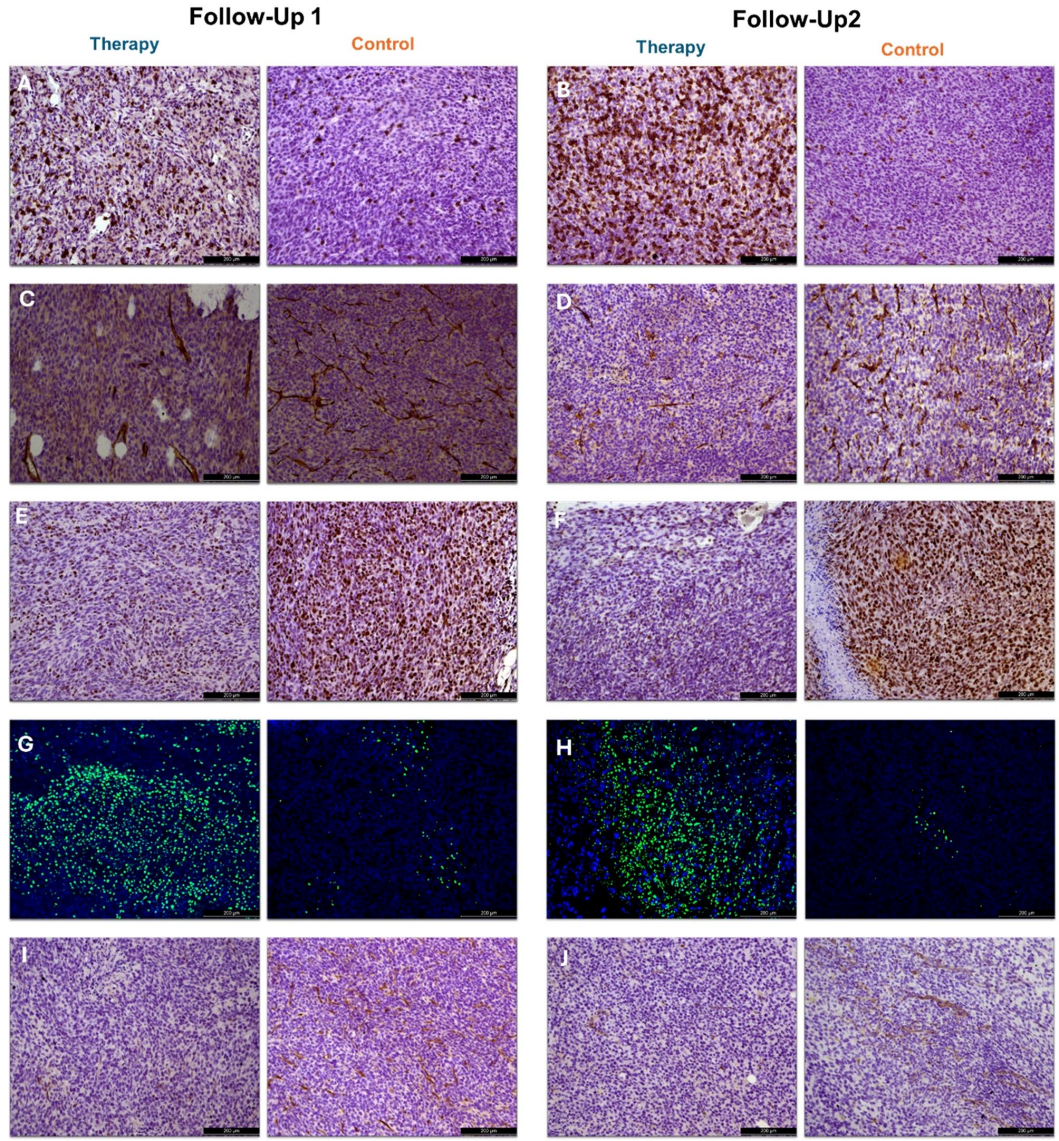
Representative immunohistochemical stainings. Representative immunohistochemical and immunofluorescence images of CT26 MSS colorectal carcinoma cells at follow-up 1 and follow-up 2 in therapy and control groups. Stainings include CD8 (tumor-infiltrating lymphocytes; **A**, **B**), CD31 (microvascular density; **C**, **D**), Ki-67 (cell proliferation; **E**, **F**), TUNEL (apoptosis; **G**, **H**), and VEGFR2 (**I**, **J**). Tumors from the therapy group show significantly higher CD8 T-cell infiltration and apoptotic activity, alongside significantly lower microvessel density, tumor cell proliferation, and VEGFR2 expression compared to controls. Scale bars = 200 μm. Representative images were selected from viable tumor regions used for quantitative analysis. Areas of extensive necrosis, tissue-processing artifacts, and section borders were excluded

Quantitative ex vivo immunohistochemical analyses were performed at follow-up 1 and follow-up 2 in an independent histological cohort (Fig. 6). At follow-up 1, Ki-67 expression was significantly lower in treated tumors (53.6 ± 21.1%) than in controls (89.5 ± 6.1%; *p* = 0.004). This difference persisted at follow-up 2, with lower proliferation in the therapy group (68.7 ± 21.6%) compared to controls (94.3 ± 5.3%; *p* = 0.035). Tumor cell apoptosis (TUNEL staining) was significantly higher in the therapy group at both follow-up 1 (59.1 ± 10.8% vs. 5.5 ± 5.1%; *p* < 0.001) and follow-up 2 (61.7 ± 15.7% vs. 8.6 ± 7.3%; *p* = 0.001). At follow-up 1, CD8 cell counts were significantly higher in the therapy group (2,369 ± 803) than in controls (684 ± 230; *p* < 0.001), and this difference remained significant at follow-up 2 (5,143 ± 1,928 vs. 841 ± 60; *p* < 0.001). Microvascular density (CD31 staining) was lower in the therapy group at both follow-up 1 (348.6 ± 59.6 vs. 1,502.1 ± 434.1; *p* < 0.001) and follow-up 2 (661.6 ± 129.3 vs. 1,555.6 ± 381.7; *p* < 0.001). Similarly, VEGFR2 expression was significantly lower in the therapy group at follow-up 1 (158.6 ± 64.0 vs. 525.6 ± 262.1; *p* = 0.001) and follow-up 2 (168.6 ± 61.8 vs. 431.0 ± 94.6; *p* = 0.001). To assess potential tissue effects of repeated CEUS examinations, the necrotic tumor fraction was compared between CEUS-imaged and non-imaged tumors at endpoint. No significant difference was observed between groups (22.2 ± 5.6% vs. 27.0 ± 15.1%; *p* = 0.755), indicating that repeated CEUS did not measurably affect tumor necrosis (Supplementary Figure S1).

## Discussion

This study demonstrates that multiparametric VEGFR2-targeted CEUS enables noninvasive detection of early vascular changes during dual immune checkpoint inhibition in murine colorectal cancer. Treated tumors showed significantly lower perfusion and VEGFR2-targeted signal compared with controls. Histology confirmed these findings, revealing lower proliferation, higher apoptosis, higher CD8 infiltration, and lower microvessel density and endothelial VEGFR2 expression.

### Tumor area dynamics and the need for early functional imaging

At follow-up 1, tumor area did not differ significantly between therapy and control groups, whereas a significantly lower tumor area was observed at follow-up 2. This pattern reflects the well-recognized delay in measurable size response frequently observed under ICI. Clinically, objective size-based responses to ICIs often emerge only after several weeks to months. In metastatic melanoma, for example, objective responses within the first three months of PD-1-based therapy occur in fewer than 10% of patients [19]. Early imaging may therefore demonstrate apparent disease stability before subsequent regression [32]. To account for these atypical response patterns, iRECIST recommends confirmatory imaging 4–8 weeks later to distinguish true progression from delayed response [33]. Large phase III trials across tumor entities further illustrate this dynamic. In nivolumab-treated clear-cell renal cell carcinoma, imaging was performed at baseline and every eight weeks, with a median time to response of 3.5 months among responders [34]. Similarly, in first-line advanced NSCLC with high PD-L1 expression treated with pembrolizumab, imaging was conducted every nine weeks, and the median time to response was 2.2 months [35]. These clinical observations highlight the need for imaging biomarkers capable of detecting early therapy-associated changes before macroscopic differences in tumor growth become apparent. In this context, CEUS enables real-time quantification of tumor perfusion and vascular parameters that may be more sensitive to early ICI effects than diameter-based measurements. Consistent with this concept, our data demonstrate that although significant differences in tumor area emerged only at later time points (follow-up 2), CEUS detected early therapy-associated vascular changes shortly after treatment initiation (follow-up 1).

### CEUS parameters correlated with histologic changes

CEUS perfusion metrics and IHC angiogenesis markers were consistently lower in the therapy group than in controls, demonstrating anti-angiogenic effects of dual ICI on the tumor vasculature. WiAUC, reflecting tumor blood flow and volume, was significantly lower in treated tumors, indicating impaired perfusion. This finding corresponded with a significantly lower microvascular density as assessed by CD31 staining. Similar patterns have been observed with direct anti-angiogenic agents: in a VEGFR2-targeted CEUS study monitoring regorafenib, WiAUC was significantly lower in treated tumors compared to controls, with CD31 staining showing > 60% lower CD31 expression [36]. However, unlike regorafenib, which directly inhibits VEGFR signaling, dual ICI modulates tumor vasculature indirectly through immune-mediated mechanisms, including enhanced CD8⁺ T-cell activity (Fig. 1B). Likewise, late-phase CEUS signal intensity (SI_8mi_n and SI_10min_), reflecting VEGFR2-bound microbubbles, was significantly lower in the therapy group, paralleling the significantly lower endothelial VEGFR2 expression observed histologically. To minimize confounding by tumor necrosis, ROIs were placed in peripheral viable tumor areas. Although microscopic necrosis cannot be entirely excluded, the close correspondence between CEUS metrics and histological findings supports a biological effect rather than a necrosis-driven lower signal. Collectively, these findings underscore the capacity of VEGFR2-targeted CEUS to noninvasively detect early therapy-induced vascular changes in colorectal cancer. The novelty of the present study does not lie in the biological role of VEGFR2 signaling itself, which has been extensively characterized, but in the longitudinal application of VEGFR2-targeted CEUS for noninvasive monitoring of immunotherapy-induced vascular changes in MSS colorectal cancer.

### VEGF-VEGFR2 signaling and therapy-induced vascular changes

The observed vascular changes are consistent with established interactions between angiogenesis and tumor immunology. VEGF-VEGFR2 signaling promotes disorganized, leaky vasculature that fosters tumor growth and impairs immune infiltration [37–39]. VEGF suppresses anti-tumor immunology by inhibiting dendritic cell maturation, polarizing macrophages, expanding regulatory T cells and myeloid-derived suppressor cells, and impairing CD8 T-cell function [40]. It also promotes T-cell exhaustion by upregulating PD-1 [8, 41]. Blocking VEGF/VEGFR2 can normalize vessels and improve T-cell access. Preclinical models have shown that combining VEGFR2 and PD-1 blockade enhances immune infiltration and tumor control [42, 43]. These studies demonstrated improved vascular functionality and reduced immunosuppressive signaling within the tumor microenvironment, thereby facilitating more effective T-cell infiltration and anti-tumor activity. While these studies used anti-angiogenic strategies, our findings suggest that dual ICI may induce comparable vascular changes indirectly through immune-mediated mechanisms. BR55 microbubbles are confined to the intravascular compartment due to their size and do not extravasate into the interstitium. They bind to VEGFR2 expressed on the luminal surface of endothelial cells, enabling intravascular molecular imaging of tumor angiogenesis [44]. Because BR55 remains intravascular and does not bind tumor cells directly, the observed lower CEUS signal reflects vascular rather than tumor cell-associated changes. Prior studies confirm that BR55 CEUS signal correlates with VEGFR2 expression and vessel density [45]. Our results indicate a significantly lower tumor perfusion accompanied by significantly higher CD8 infiltration, suggesting a more immunologically permissive tumor microenvironment. Recent work has demonstrated that therapy-induced vascular functional changes improve perfusion and alleviate hypoxia within the tumor microenvironment, promoting CD8 T-cell infiltration and potentiating ICI therapy efficacy [46]. These observations provide a mechanistic framework that is consistent with the higher CD8 T-cell infiltration observed in our study. Whether the observed reductions in perfusion and vessel density were accompanied by altered tumor hypoxia was not specifically assessed in the present study. This aspect warrants cautious interpretation, as HIF1α is a dynamic endogenous hypoxia marker that may be influenced by tissue sampling, pre-analytical conditions, and intratumoral heterogeneity. Furthermore, the relationship between reduced vessel density, lower perfusion signals, and tumor hypoxia during immunotherapy remains complex and cannot be inferred directly from the present data, as immune-mediated vascular remodeling and vascular normalization may alter tissue oxygen delivery despite reductions in angiogenic signaling [17]. Previous studies have demonstrated that vascular normalization can improve tissue oxygenation and antitumor immunity despite reduced angiogenic signaling [17, 42]. Future studies incorporating prospectively standardized tissue sampling and dedicated hypoxia-focused analyses, including markers such as HIF1α and exogenous hypoxia probes, may further elucidate the interplay between vascular remodeling, tumor oxygenation, and immune activation.

### Translational implications of VEGFR2-targeted CEUS

CT26 is a MSS murine colorectal tumor model that recapitulates key features of human MSS CRC, including a relatively low baseline T-cell infiltration and limited responsiveness to PD-1/PD-L1 inhibitor monotherapy [47]. Clinically, MSS colorectal cancers typically exhibit poor responses to single-agent ICI due to immune exclusion and low intrinsic immunogenicity [1, 2]. Although CT26 tumors are MSS, they retain conditional immunogenicity: when sufficient T-cell priming and expansion are achieved, robust CD8 T-cell-mediated tumor rejection can occur, largely driven by responses against the immunodominant AH1 rejection antigen [4]. This feature explains the apparent paradox that CT26 displays limited sensitivity to PD-1/PD-L1 monotherapy, yet responds more effectively to combined ICI. Dual PD-(L)1 and CTLA-4 inhibition enhances both T-cell priming and effector function, overcoming inhibitory signaling at complementary stages of the anti-tumor immune response. As demonstrated by Fiegle et al. [26], combined blockade in CT26 results in significantly higher tumor regression compared with either monotherapy, consistent with the concept that coordinated ICI can surpass the activation threshold required to generate effective anti-tumor immunity in MSS tumors. Importantly, CT26 is a syngeneic, immunocompetent tumor model in BALB/c mice [48], providing a physiologically intact immune system for investigating therapy-associated vascular changes under ICI. In the present study, CEUS signal intensity and perfusion parameters were significantly lower in treated tumors, and corresponding histologic findings confirmed lower VEGFR2 expression, lower microvessel density, and higher CD8 infiltration. These data indicate that dual ICI induces therapy-associated vascular changes accompanied by immune activation in CT26 tumors, supporting the utility of VEGFR2-targeted CEUS for monitoring immunotherapy-induced vascular dynamics in MSS colorectal cancer. Importantly, additional histological assessment revealed no significant difference in necrotic tumor fraction between CEUS-imaged and non-imaged tumors. Although histological validation was intentionally performed in a separate cohort to avoid potential interference of prior BR55 administration with VEGFR2 immunohistochemistry, these findings provide further support that repeated low-mechanical-index CEUS examinations did not induce detectable alterations in tumor viability. This observation strengthens the translational relevance of VEGFR2-targeted CEUS as a noninvasive imaging approach for longitudinal monitoring of therapy-associated vascular changes.

### Limitations

The relatively small sample size, typical for murine studies, may limit statistical power and generalizability. ROI placement was restricted to viable tumor regions; however, microscopic necrosis or intratumoral perfusion heterogeneity may have influenced measurements. Advanced CEUS techniques, such as volumetric or higher-resolution imaging, may improve spatial accuracy. Only selected immune and vascular markers were assessed; broader immunophenotyping could provide more comprehensive insights. Although CT26 is relatively resistant to PD-1/PD-L1 monotherapy, it responds to dual immune checkpoint inhibition and serves as a relevant model for immune activation. Attrition occurred in both study arms due to predefined humane endpoints, anesthesia-related complications, or technical imaging failure, which may have introduced a degree of selection bias despite the overall balanced distribution between groups. Histological validation was performed in an independent cohort; therefore, direct animal-level correlations between imaging and histological findings were not possible. To address potential effects of repeated imaging, we additionally compared necrotic tumor fractions between CEUS-imaged and non-imaged tumors and found no significant differences; however, subtle biological effects not captured by necrosis assessment cannot be entirely excluded. In addition, non-targeted control microbubbles were not included; therefore, a residual contribution of nonspecific microbubble retention within tortuous tumor vasculature cannot be fully excluded. However, late-phase signal analysis after clearance of freely circulating microbubbles and concordant ex vivo reductions in VEGFR2 expression and microvascular density support a predominantly target-related signal contribution. Clinical translation remains challenging, as longitudinal CEUS monitoring during immunotherapy has not yet been established, and lesion accessibility may limit applicability.

## Conclusion

VEGFR2-targeted CEUS tracked longitudinal tumor response to dual anti-PD-L1/anti-CTLA-4 immunotherapy in CRC. Treated tumors showed significantly lower perfusion and VEGFR2-targeted binding, paralleled by significantly lower vessel density and VEGFR2 expression, along with significantly higher CD8 infiltration and apoptosis. These results support a link between therapy-associated vascular changes and immune activation. CEUS thus represents a promising noninvasive imaging approach for monitoring early immunotherapy-associated vascular changes and warrants further evaluation as a translational imaging biomarker.

## Supplementary Information

Below is the link to the electronic supplementary material.

Supplementary Material 1

Supplementary Material 2

## Data Availability

The datasets used and/or analysed during the current study are available from the corresponding author on reasonable request.

## Abbreviations

CRC: Colorectal Cancer
ICI: Immune Checkpoint Inhibition / Inhibitors
MSS: Microsatellite Stable
MSI: Microsatellite Instable
VEGF: Vascular Endothelial Growth Factor
VEGFR2: Vascular Endothelial Growth Factor Receptor 2
CEUS: Contrast-Enhanced Ultrasound
PD-L1: Programmed Death-Ligand 1
CTLA-4: Cytotoxic T-Lymphocyte-Associated Protein 4
CD8: Cluster of Differentiation 8
Ki-67: Marker of Cellular Proliferation
TUNEL: Terminal Deoxynucleotidyl Transferase dUTP Nick End Labeling
CD31: Cluster of Differentiation 31
ROI: Region of Interest
TIC: Time-Intensity Curve
WiAUC: Wash-in Area Under the Curve
SI_8min_: Signal Intensity at 8 min
SI_10min_: Signal Intensity at 10 min
