# Supplementary material for: CEUS with VEGFR2-targeted microbubbles for monitoring of early immunotherapy effects in a colorectal cancer model
Source: Cancer Imaging. 2026 Jul 31;26:94. doi: 10.1186/s40644-026-01101-0 (PMC13428429; doi:10.1186/s40644-026-01101-0)
Supplement: Supplementary file 1 — Supplementary Material 1 [file 40644_2026_1101_MOESM1_ESM.docx]

**Supplementary Figure S1. Representative H&E-stained tumor sections from CEUS-imaged and non-imaged (IHC) used for necrosis assessment.** Necrotic regions were identified morphologically by loss of nuclear staining and eosinophilic cellular debris. Quantitative analysis revealed no significant difference in necrotic tumor fraction between CEUS-imaged and non-imaged tumors (22.2 ± 5.6% vs. 27.0 ± 15.1%; p = 0.755). Scale bars = 400 μm. Representative images were selected from viable tumor regions used for quantitative analysis. Areas of tissue-processing artifacts and section borders were excluded.
