# Supplementary figures and images for: CEUS with VEGFR2-targeted microbubbles for monitoring of early immunotherapy effects in a colorectal cancer model
Source: Cancer Imaging. 2026 Jul 31;26:94. doi: 10.1186/s40644-026-01101-0 (PMC13428429; doi:10.1186/s40644-026-01101-0)

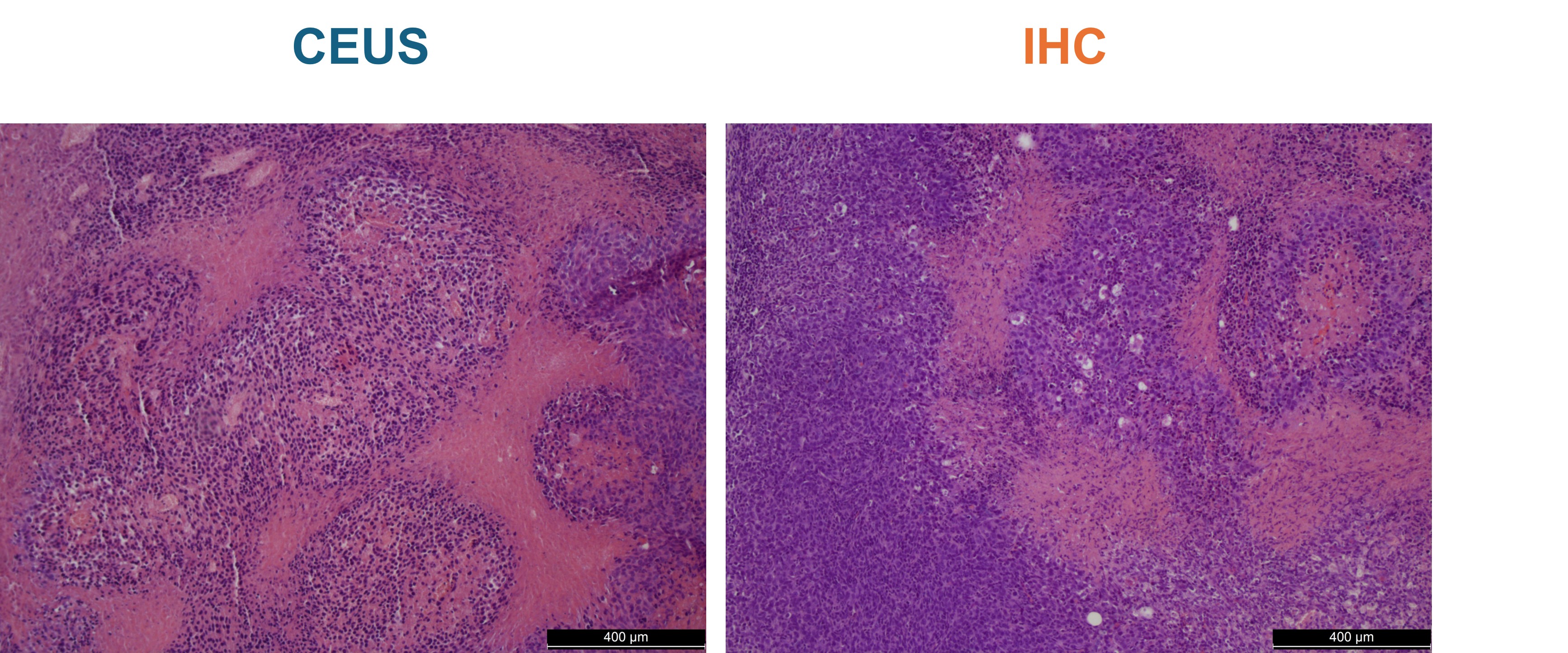

Supplement: Supplementary file 2 — Supplementary Material 2 [file 40644_2026_1101_MOESM2_ESM.jpg]
